# 10.5 T In Vivo Head Imaging With Universal RF Shimming

**DOI:** 10.1002/mrm.70262

**Published:** 2026-01-19

**Authors:** Young Woo Park, Simon Schmidt, Wolfgang Bogner, Gregory J. Metzger, Małgorzata Marjańska

**Affiliations:** ^1^ Center for Magnetic Resonance Research, Department of Radiology University of Minnesota Minneapolis Minnesota USA; ^2^ Henry M. Jackson Foundation for the Advancement of Military Medicine, Inc Bethesda Maryland USA; ^3^ Department of Radiology Uniformed Services University of the Health Sciences Bethesda Maryland USA; ^4^ Medical Physics in Radiology German Cancer Research Center (DKFZ) Heidelberg Germany; ^5^ High Field MR Center, Department of Biomedical Imaging and Image‐Guided Therapy Medical University of Vienna Vienna Austria; ^6^ Christian Doppler Laboratory for MR Imaging Biomarkers (BIOMAK) Vienna Austria; ^7^ Comprehensive Center for AI in Medicine (CAIM) Medical University of Vienna Vienna Austria

## Abstract

**Purpose:**

Brain MR imaging at 10.5 T ultra‐high field offers significant improvements in signal‐to‐noise ratio (SNR), but faces challenges with B_1_^+^ inhomogeneity. Parallel‐transmission (pTx) can be used to achieve a more uniform RF field distribution, but necessitates the use of B_1_^+^ calibration in the region of interest. This study explores a universal B_1_^+^ shim solution on 10.5 T that could eliminate the need for time‐consuming subject‐specific B_1_^+^ calibration.

**Methods:**

B_1_^+^ data from 7 participants (19 sessions) were used to develop the universal B_1_^+^ shim, which was then validated against traditional subject‐specific approaches using T_1_‐weighted MP2RAGE structural images in 5 participants (6 sessions). Statistical comparisons of tissue and subcortical segmentations were conducted using popular neuroimaging tools SPM and FreeSurfer, respectively.

**Results:**

The universal shim rapidly converged with a small training dataset, likely due to consistent positioning and the simplicity of B_1_^+^ shimming used for head imaging. Whole‐brain tissue segmentation showed no statistically significant differences between universal and subject‐specific solutions, with only minor variations near the ventricles and inferior brain regions in the detailed subcortical segmentation. The proposed universal B_1_^+^ shim reduces examination time by removing the need for separate data acquisition and optimization.

**Conclusion:**

These findings suggest that the universal B_1_^+^ shim is a viable substitute for subject‐specific approaches, offering a more efficient solution for neuroimaging applications. Additionally, it confirms that 10.5 T MRI can produce reliable structural brain imaging data, paving the way for broader adoption of ultra‐high field MRI in neuroimaging research.

## 1 Introduction

Brain MR imaging at ultra‐high field (UHF) 10.5 T offers the potential for significant gains in signal‐to‐noise ratio (SNR) over established 7 T systems, promising improvements in spatial, spectral, and temporal resolutions [1]. Recent advancements in head coils aimed at achieving the ultimate intrinsic SNR [2] with high transmit (Tx) and receive (Rx) channel counts for imaging at 10.5 T [3, 4] have demonstrated the feasibility of high‐resolution, whole human brain imaging at these elevated fields. These capabilities, once achievable only in nonhuman primate studies [5, 6], now enable detailed investigations of intricate anatomical structures [3], high‐fidelity functional activation patterns [4], and improved identification of neurochemicals in the human brain [7].

However, compared to 7 T, the inherent challenges of Tx radiofrequency (RF) field (B_1_^+^) inhomogeneity become more pronounced at 10.5 T due to the shorter RF wavelength. As a result, optimized and efficient RF management strategies are even more critical for effective head imaging at such extreme field strengths. One such strategy is parallel‐transmission [8] (pTx), a technique employing multiple independent transmit channels driving several elements on a pTx enabled coil which can be used to achieve a more uniform RF field distribution. Despite the development of numerous different pTx approaches, a significant bottleneck remains: the subject‐specific nature requiring time‐consuming B_1_^+^ mapping and subsequent optimization of RF transmit parameters, analogous to B_0_ shimming. Furthermore, while increasing the number of Tx channels enhances the degrees of freedom and overall power delivery for pTx, the larger number of Tx channels also intensifies B_1_^+^ field interactions, thereby complicating the achievement of a spatially uniform excitation. Finally, this process, which in our case involves manually copying the large data from the scanner to external computer, manually drawing the shimming regions, then copying the shim values back to the scanner, can add several minutes to the scanning protocol, which could significantly affect the overall data acquisition efficiency.

To address this limitation and enhance throughput at 10.5 T, we investigated strategies to eliminate the B_1_^+^ calibration process. One promising technique is the recently proposed universal pulses concept [9], which leverages a diverse database of RF field maps to generate optimized, subject‐independent pTx solutions. Universal RF shimming has been demonstrated in various UHF applications on a clinical 7 T systems with 8 Tx coils for brain fMRI [10], CEST [11], MRS [12], and body imaging [13, 14]. Universal RF shimming with even higher field strengths (9.4 T or above) and channel count (16 Tx) has also been reported recently [15, 16].

A circularly polarized (CP) mode based on the azimuthal layout of the 16 transceiver elements (two axial rows of eight, offset by 22.5°) yielded a suboptimal B_1_^+^ distribution. The poor performance of CP mode motivated the use of B_1_ shimming optimized for central power efficiency in a head‐shaped phantom in Waks et al. [3] and the development of the universal pTx solution presented in this work.

In this manuscript, we detail the development of a universal B_1_^+^ shim solution for use with our 16 Tx/Rx + 64 Rx channel head coil [3] at 10.5 T. We evaluate the feasibility of the universal solution by comparing its imaging performance to subject‐specific B_1_^+^ shims derived from individual RF field maps to assess how closely the universal solution resembles subject‐specific solution. Finally, we discuss the strengths and weaknesses of both methods, as well as the potential implications of the proposed approach for UHF MRI beyond 10.5 T.

## 2 Methods

### 2.1 Hardware Specification

All experiments were performed on a 10.5 T Siemens MAGNETOM MRI scanner (software version VE12U‐AP01‐F50, Siemens Healthineers, Erlangen, Germany) featuring an 88 cm bore, passively shielded whole‐body magnet, and a whole‐body gradient system with a SC72D gradient coil with a patient accessible bore diameter of 60 cm. RF transmission and reception were achieved using the 16 Tx/Rx + 64 Rx channel pTx head coil [3] driven by 16 independent 2 kW power amplifiers (Stolberg HF‐Technik AG, Stolberg, Germany) and 80 of the 128 available receivers.

All imaging procedures were conducted under an FDA approved Investigational Device Exemption with the approval from the Institutional Review Board at the University of Minnesota. Participants were medically screened and cleared for participation in the study, provided written informed consent prior to imaging, and underwent vestibular, physiologic, and sensorimotor assessment before and after magnetic field exposure [17].

### 2.2 Universal B_1_ ^+^ Shim Solution Derivation

**FIGURE 1:**
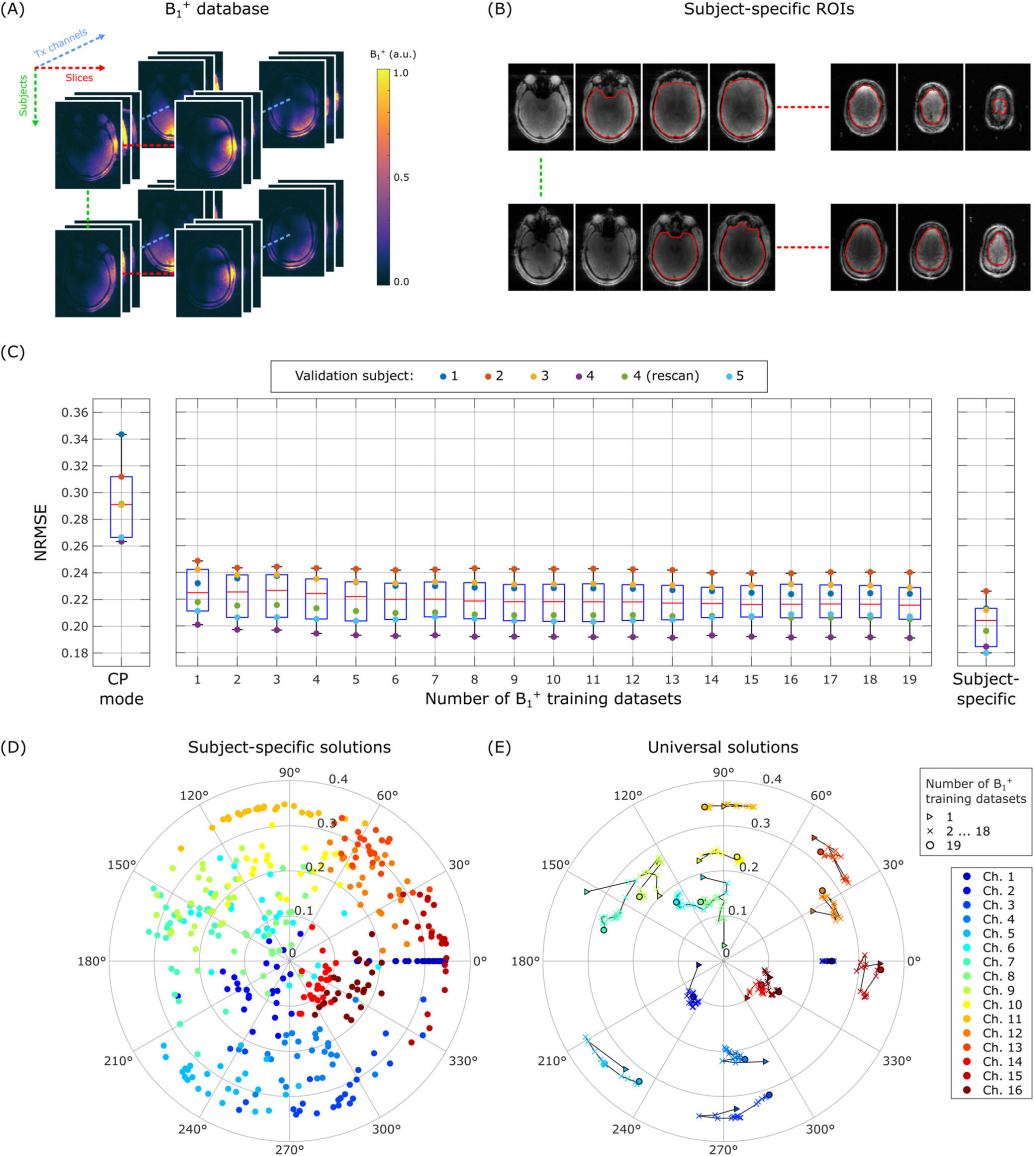
Optimization scheme for generating a universal shim solution at 10.5 T and its characteristics. (A) First, the database of B_1_^+^ field maps for training was populated using 19 datasets; (B) second, regions‐of‐interest (ROI) maps, which encompassed the supraorbital cerebrum, were manually defined for all B_1_^+^ datasets; (C) finally, a universal solution was derived by jointly optimizing the L2‐norm of the subject‐specific objective functions across all 19 training datasets; (D) Magnitude and phase of the subject‐specific solutions of all 19 training and 6 validation datasets; (E) Magnitude and phase of the universal solution for an increasing number of training datasets.

Relative channel‐wise complex B_1_^+^ data [18] were initially acquired from seven participants (3 females and 4 males; age range 23–74 years; median age 42 years) who underwent multiple scans (*N* = 19). Data acquisition employed a 2D multi‐slice RF spoiled gradient echo sequence with TR/TE = 100/2.2 ms, nominal FA = 15°, and resolution = 2.0 × 2.0 × 4.0 mm^3^ over 15 transversal slices (112 mm head‐foot coverage) with an acquisition time of 1:29 min. Regions of interest (ROIs) encompassing the supraorbital cerebrum were manually defined for all B_1_^+^ datasets and subsequently used for pTx optimization (Figure 1A,B). The ROIs were chosen specifically for our study focusing on the MRSI in the cortical regions superior to the cerebellum [7]. Phase‐magnitude B_1_^+^ shimming was performed by minimizing a nonlocalized efficiency cost‐function [19] with constraints on over‐ and under‐flipping ($\alpha_{\max}$ = 1.3; $\alpha_{\min}$ = 0.8) and channel‐wise maximal normalized shimming weights (< 0.35). These weights were normalized such that the L2‐norm of the shimming vector equals one (i.e., normalized to total power). The upper limit of 0.35 was chosen to constrain the power of a single channel to twice that of a phase‐only solution. A universal solution was derived by jointly optimizing the L2‐norm of the subject‐specific objective functions across all 19 training datasets [20].

### 2.3 Universal B_1_ ^+^ Shim Solution Evaluation

The universal B_1_^+^ shim solution derived from the full training database was validated in an independent cohort of five individuals (4 males and 1 female; age range 29–64 years; median age 55 years), with one participant undergoing a second scan several weeks later (*N* = 6). Relative channel‐wise B_1_^+^ data were acquired to calculate subject‐specific B_1_^+^ shimming solutions using the optimization approach described above. Absolute B_1_^+^ maps, acquired with a pre‐saturated turbo flash sequence [21] (TR/TE = 7000/1.3 ms, FA = 15°, resolution = 2.0 × 2.0 × 5.0 mm^3^, 15 slices, acquisition time = 15 s), were used for power calibration of both subject‐specific and universal solutions. Subsequently, T_1_‐weighted MP2RAGE [22] data were acquired using both shim solutions with the following parameters: TR/TE = 5000/2.04 ms, TI_1_/TI_2_ = 800/2920 ms, FA_1_/FA_2_ = 6°, resolution = 1.0 × 1.0 × 1.0 mm^3^, and GRAPPA acceleration factor 3 with 48 auto‐calibration signal (ACS) lines with a total acquisition time of 7:07 min.

Convergence of the universal solution was investigated by iteratively calculating universal solutions for an expanding database. Starting with only the first acquired dataset, we incrementally added the remaining 18 training datasets in their acquisition order. Each of these intermediate universal solutions was then validated on the six validation datasets (five independent participants). This validation involved applying the resulting B_1_^+^ shim solution to the channel‐wise B_1_^+^ maps of the validation participants and subsequently calculating the normalized root mean squared error (NRMSE) across subject‐specific ROIs. Performance of the universal solution was compared to a CP mode defined solely based on the azimuthal position of the 16 transceiver elements, subject‐specific B_1_^+^ shimming solutions, phase only B_1_^+^ shimming, and B_1_^+^ shimming with the coefficient of variation as a cost function, and efficiency‐based shim for the whole brain for the supraorbital cerebrum and the whole brain.

### 2.4 Image Analysis

**FIGURE 2:**
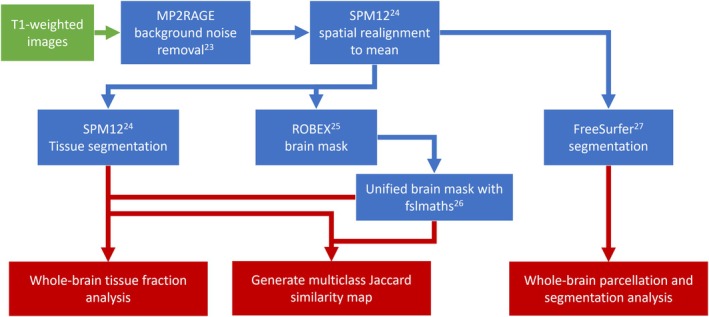
Post‐processing scheme for performing tissue segmentation on 10.5 T MP2RAGE data with popular neuroimaging scripts and processing tools. Superscripted numbers denote the corresponding citations for the referenced software packages.

Post‐processing of the anatomic imaging data was performed using standard neuroimaging analysis tools (Figure 2). MP2RAGE data underwent denoising [23] to eliminate the background noise. Both universal and subject‐specific solutions were aligned to their mean position and segmented using SPM12 [24]. A unified brain mask was created by summing the two brain masks generated using ROBEX and fslmaths [25, 26].

Tissue fractions (white matter, WM; gray matter, GM; cerebrospinal fluid, CSF) were computed within the unified brain mask, and the results were compared between the two B_1_^+^ shim solutions using a paired, two‐tailed *t*‐test. To visualize the differences in tissue fractions, the Multiclass Jaccard Similarity (MJS) map was used. For each voxel, the MJS was calculated as:

$$\sum_{n=1}^{N}w_{n}\frac{\left(A_{n}\cap B_{n}\right)}{\left(A_{n}\cup B_{n}\right)}$$

where *N* is the number of tissue classes (3); *n* denotes the tissue type; $w_{n}$ is the weight of the tissue class (computed by the mean fraction); $A_{n}$ and $B_{n}$ are estimated tissue fractions for tissue type *n*; and the ∩ and ∪ operator returns the smaller and larger tissue fraction, respectively. The resulting MJS ranges from 0 (no overlap) to 1 (identical composition). For better visualization, the results were plotted as “1 – MJS” to highlight voxels with large differences.

Aligned brain images were automatically parcellated and segmented using FreeSurfer [27]. The volumes of each cortical and subcortical region were extracted and compared using the paired two‐tailed *t*‐test. For those regions with symmetrical pairs, the asymmetry index (AsI) [28] was computed as:

$$\mathrm{AsI}=\frac{\left(V_{\text{left}}-V_{\text{right}}\right)}{\left(V_{\text{left}}+V_{\text{right}}\right)}$$

where *V*_*left*_ and *V*_*right*_ correspond to the volumes of the left and right hemispheres, respectively. AsI values from two images were also compared using a paired two‐tailed *t*‐test.

## 3 Results

All participants successfully underwent scanning at 10.5 T, reporting no adverse effects. The universal B_1_^+^ shim solution was calculated offline using the 19 training datasets prior to the validation imaging. In contrast, subject‐specific B_1_^+^ shimming for the 6 validation datasets necessitated acquisition of B_1_^+^ maps (1:29 min), export of the raw data (30 s), reconstruction of RF field maps (4:30 min), manual ROI definition (5 min), subsequent pTx optimization (3 min), and data transfer (20 s), requiring approximately 15 min per subject. Consequently, T_1_‐weighted images acquired with the universal B_1_^+^ shim were obtained concurrently with the subject‐specific shim optimization process.

### 3.1 Convergence of Universal B_1_ ^+^ Shim Solution

Figure 1C shows the NRMSE across subject‐specific regions of interest (ROIs) in six validation datasets for all intermediate universal solutions up to the final universal solution based on all 19 training datasets, as well as for the CP mode and subject‐specific solutions. The median NRMSE decreased from 22.5% for the universal solution based on the first training dataset to 21.6% for the final universal solution incorporating all 19 datasets. For comparison, the median NRMSE achieved with the CP mode was 29.1%, while the subject‐specific solutions yielded 20.4%. Figure 1D shows the magnitude and phase of the subject‐specific solution of all 19 training and six validation datasets for each coil channel. Figure 1E shows the magnitude and phase of the universal solution for an increasing number of training datasets for each coil channel and shows the rapid convergence of the universal solution.

**FIGURE 3:**
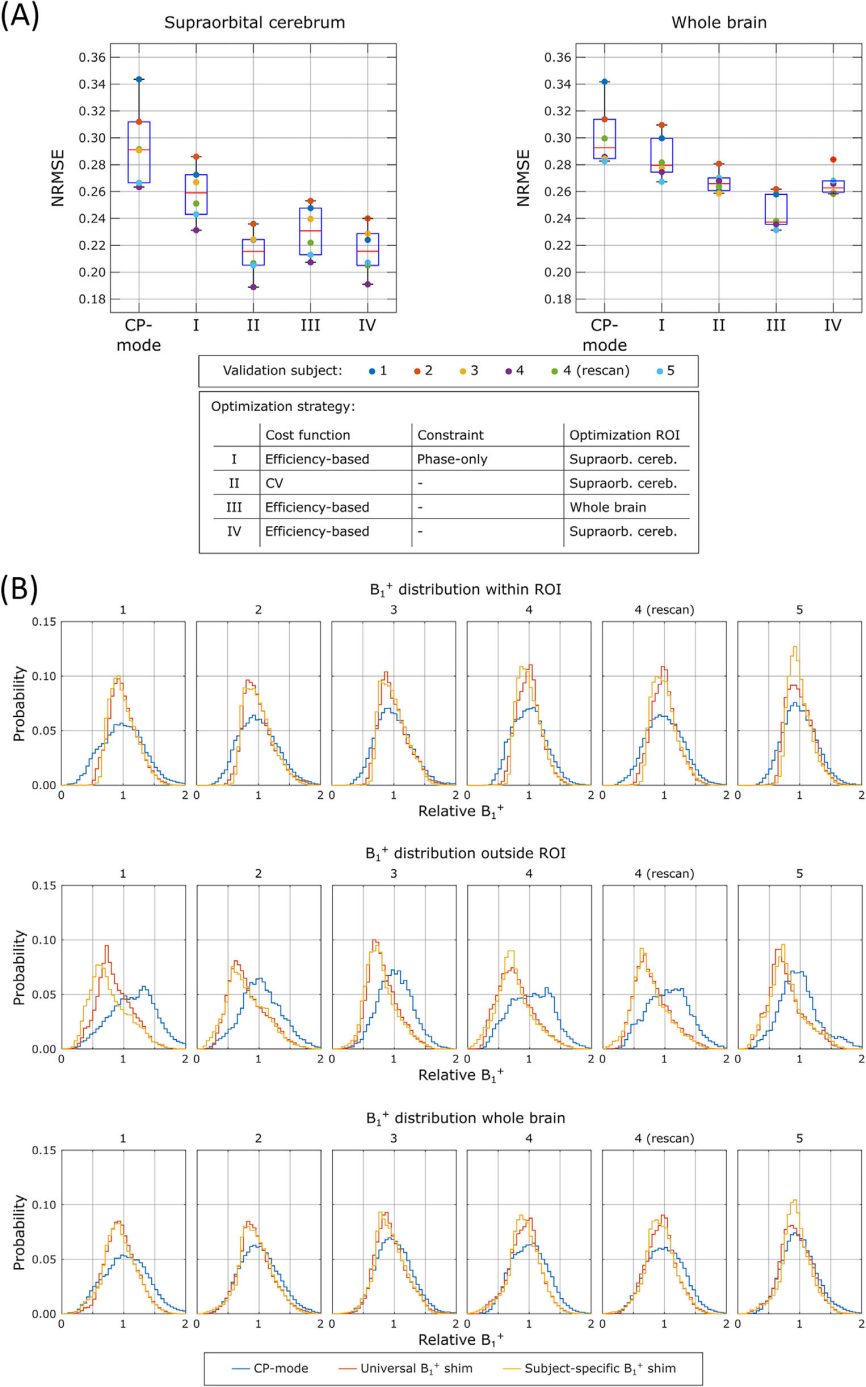
Performance comparison in six validation datasets. (A) Summary of optimization outcomes within the target region‐of‐interest (ROI: supraorbital cerebrum, left) and across the whole brain (right). I versus IV: Use of a phase‐magnitude solution rather than phase‐only adds substantially to the performance improvement of the universal solution over the CP‐mode. II versus IV: The efficiency‐based cost function performs almost identical to that using the coefficient of variation. III versus IV: As expected, optimization within the ROI yields better performance. (B) Illustration of the B_1_^+^ distributions within the optimization ROI (top), outside of the ROI (middle), and across the whole brain (bottom) for the CP‐mode, the universal solution, and the subject‐specific solutions applied to six validation datasets. Within ROI: Subject‐specific solutions perform best, followed by the universal solution, which is consistent with NRMSE trends in Figure 1. Outside ROI: The universal solution tends to have less voxels with very low B_1_^+^ values than subject‐specific solution. The universal solution, optimized over multiple datasets with slight variations in the positioning of the subjects, also is more general in terms of the spatial B_1_^+^ distributions.

Figure 3A shows the NRMSE across ROIs and the whole brain, respectively, in 6 validation datasets for different optimization strategies including phase‐only B_1_^+^ shimming (I), B_1_^+^ shimming with the coefficient of variation as a cost function (II), efficiency‐based shim for the whole brain (III), and universal solution (IV). The universal solution performed substantially better than CP mode and phase‐only B_1_^+^ shimming. The universal solution with the efficiency‐based cost function that we used performed almost identically to the B_1_^+^ shimming with the coefficient of variation as a cost function. As expected, the better performance is observed in the region for which B_1_^+^ was optimized. Figure 3B shows the distribution of the B_1_^+^ within and outside of the ROIs and for the whole brain for the CP mode, universal shims, and subject‐specific solutions for six validation datasets. The B_1_^+^ distribution is improved outside the targeted ROIs with the universal solution compared to the subject‐specific solutions in which there is a higher probability for really low relative B_1_^+^ values.

### 3.2 B_1_ ^+^ Field Homogeneity and Image Quality

**FIGURE 4:**
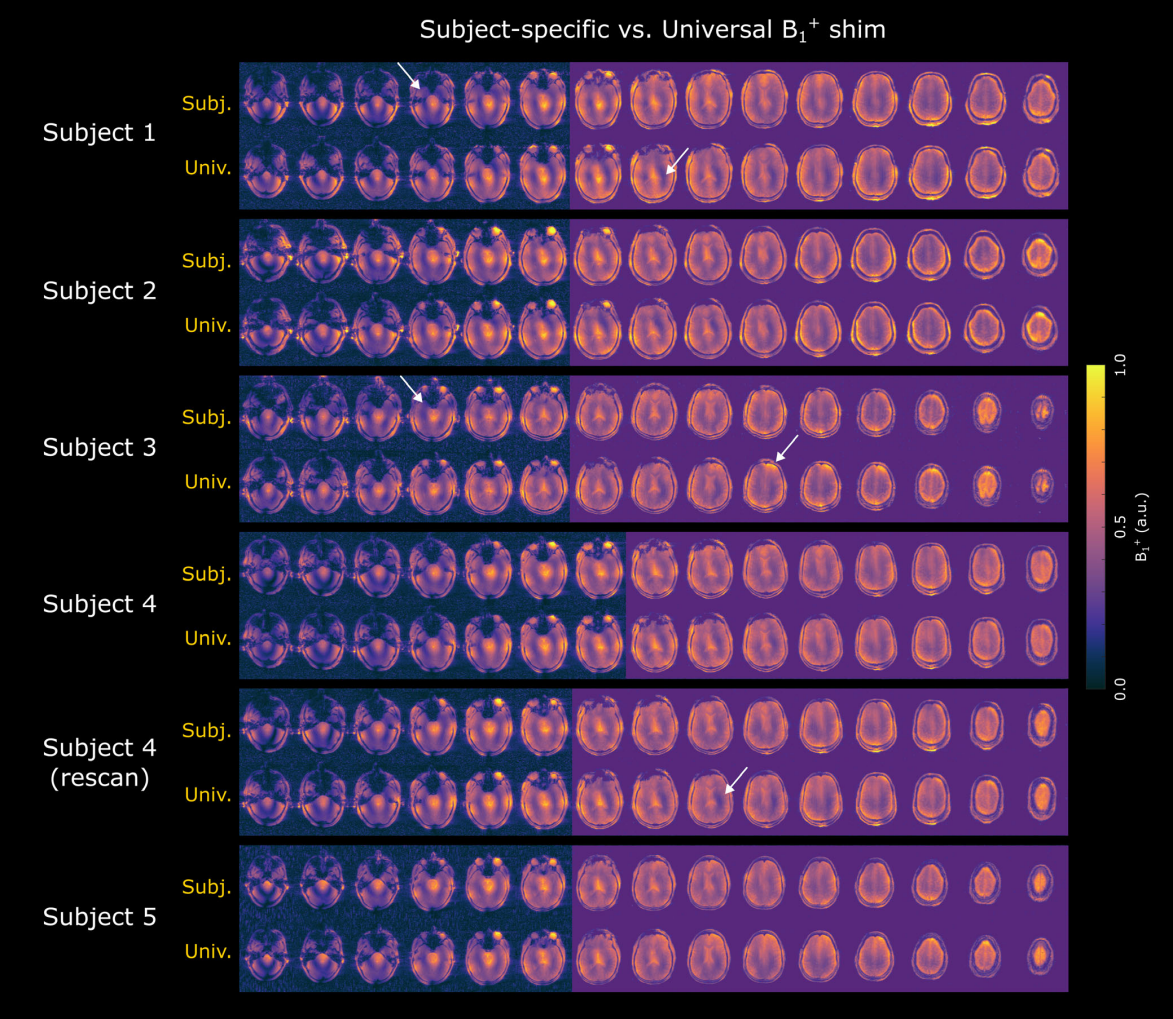
B_1_^+^ distributions for subject‐specific (top row) and universal (bottom row) shim solutions for each of the six validation datasets. The white arrows indicate representative regions of over‐ and under‐flipping. Slices with purple background denote those used for subject‐specific shim calibrations.

**FIGURE 5:**
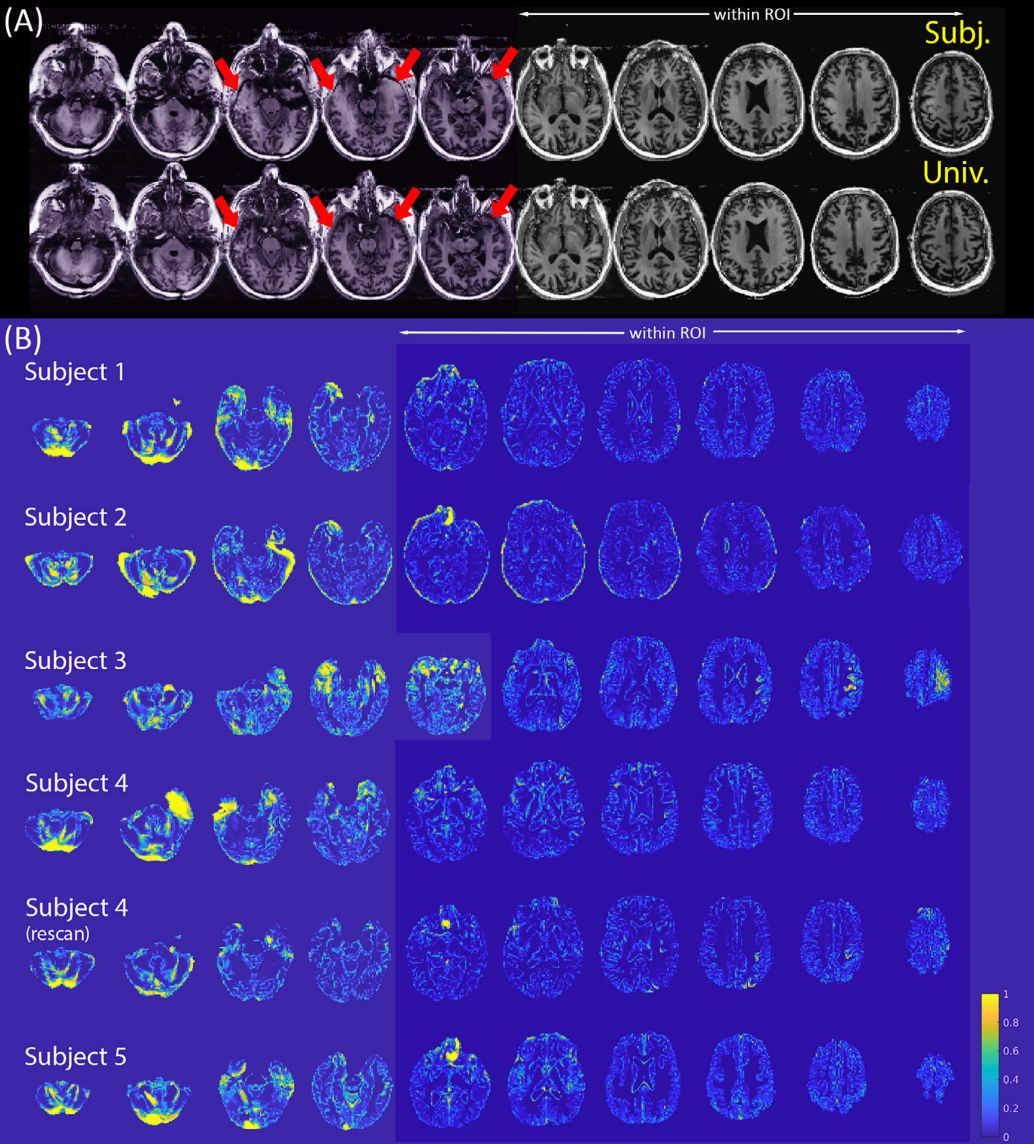
(A) Denoised MP2RAGE images obtained from subject 3 in Figure 4 with the subject‐specific (top) and universal (bottom) shim solutions. Note the loss of gray matter tissue contrasts around the highlighted regions with the subject‐specific shim solution. Slices with a purple tint denote those outside the subject‐specific shim regions. (B) Multiclass Jaccard Similarity (MJS) results highlight the brain regions showing the largest differences between the subject‐specific and universal shim solutions. Note that for visualization purposes, the values displayed in this figure are “1‐MJS” with 0 indicating identical composition and 1 no overlap. Additionally, slices with the blue background denote those inside the subject‐specific shim regions.

Visual inspection of B_1_^+^ maps (Figure 4) revealed generally similar field distributions for both subject‐specific (top rows) and universal shims. However, subject‐specific solutions exhibited subtly more uniform excitation profiles with fewer pronounced regions of over‐ and under‐flipping, as highlighted by the white arrows in Figure 4. Quantitatively, coefficients of variation computed across the whole brain did not differ significantly (*p*‐value > 0.05, paired *t*‐test) between the two shimming approaches across six validation datasets (subject‐specific: 28.3/28.4/26.2/27.1/26.3/25.2%; universal: 26.0/28.4/26.0/26.6/25.8/26.8%). Despite these subtle B_1_^+^ field variations, the use of both subject‐specific and universal shim solutions resulted in high‐quality anatomical T_1_‐weighted images (Figure 5A) deemed suitable for standard neuroimaging analysis.

### 3.3 Tissue Fractions

Whole‐brain tissue segmentation analysis of WM, GM, and CSF volumes (cross‐subject mean tissue fractions of GM/WM/CSF—subject‐specific: 55.2/33.0/11.8%; universal: 56.1/33.4/10.5%) did not differ significantly between data acquired with subject‐specific and universal shims (*p*‐values > 0.05). However, the MJS maps (Figure 5B) highlight localized discrepancies, particularly within the temporal lobes, cerebellum, and superior brain regions. These differences manifest primarily as variations in GM segmentations, suggesting variations in tissue contrast between the two B_1_^+^ shim solutions. Notably, the temporal lobes and cerebellum were outside the ROIs used for both subject‐specific and universal B_1_^+^ shim optimizations.

### 3.4 Segmentation and Asymmetry Index

Comparison of parcellation and segmentation (Table S1) results showed significant differences when not adjusted for multiple comparisons in the cortical and subcortical regions, particularly around the ventricles and the lower part of the brain. AsI analysis (Table S2) shows the values from 10.5 T were in line with a previously reported AsI range from 3 T [28] (subject‐specific: 0.36 to −0.41; universal: 0.45 to −0.36) with significant difference only seen in temporal and occipital lobes and cerebellar white matter.

## 4 Discussion

This study successfully demonstrated the feasibility and potential of a universal B_1_^+^ shim solution for human head imaging at 10.5 T using the 16 Tx/Rx + 64 Rx channel pTx coil. In vivo validation, conducted with widely used neuroimaging analysis software such as SPM and FreeSurfer, revealed no statistically significant differences in whole‐brain B_1_^+^ homogeneity or tissue segmentation between the universal and subject‐specific B_1_^+^ shim solutions. A detailed tissue parcellation and subcortical segmentation revealed that most regions exhibited no significant differences, except for a few regions near the ventricles and the inferior part of the brain. This key finding suggests that the universal shim holds promise as a viable substitute for the more time‐consuming subject‐specific approach in various neuroimaging applications. Additionally, it demonstrates that 10.5 T MRI can produce usable structural imaging data that can be processed by standard brain analysis software.

The development of an effective universal B_1_^+^ shim solution at 10.5 T presents a significant step towards streamlining UHF imaging. The rapid convergence observed in the universal B_1_^+^ shim solution with a relatively small training database is likely attributable to the consistent head positioning within the coil across participants and application of a simple B_1_^+^ shimming approach. Furthermore, the simplicity of using B_1_^+^ shimming instead of a more complex method, such as full dynamic pTx pulses, likely contributes to this rapid convergence, as the approach inherently has fewer degrees of freedom. Hence, this suggests that robust universal solutions can be derived efficiently for different imaging protocols. Future work should explore the integration of additional constraints, such as peak local SAR, into the universal solution. Currently, the universal solution was optimized solely based on the B_1_^+^ metric. By utilizing the virtual observation points (VOPs) [29] for the coil, a direct constraint on the 10 g peak local SAR could be incorporated [16], potentially enabling the derivation of a universal solution with reduced SAR. Population‐specific approach for further optimization of the universal solution, as reported by Tyshchenko et al. [30, 31], could be another venue worth exploring.

A key benefit of the universal B_1_^+^ shim solution is its substantial reduction in the examination time, estimated at about 15 min per subject, by obviating the need for B_1_^+^ mapping, and subsequent manual ROI creation and RF optimization. While there are multiple ways to accelerate the process, the demonstrated performance of the universal shim compared to subject‐specific solution make it attractive for rapid, calibration‐free MP2RAGE brain structural imaging. While the universal B_1_^+^ shim solution demonstrated promising results for MP2RAGE‐based brain segmentation at 10.5 T, subtle regional differences were observed in the MJS maps. Specifically, the data acquired with the universal solution showed potential for artifacts in the cerebellar vermis and superior brain regions, likely due to residual B_1_^+^ inhomogeneity leading to signal intensity variations that can affect tissue contrast in MP2RAGE data. Conversely, subject‐specific shims showed some limitations in the temporal lobes, which, however, were outside the target ROIs. Future work could include a comparison of the subject‐specific and universal B_1_^+^ shim solutions with an ROI covering inferior cortical regions, cerebellum, and brainstem.

A primary limitation of this study was the absence of a direct “ground truth” reference, precluding a quantitative assessment of the absolute segmentation accuracy of both shimming methods. While we explored the use of 3 T structural data for segmentation evaluation, inter‐field strength tissue contrast variations rendered this approach unreliable as previous studies have shown that segmentation results systematically differ depending on the field strengths (between 1.5, 3, and 7 T) [32, 33] as well as the type of pulse sequences (MPRAGE vs. MP2RAGE) [34, 35]. Furthermore, the in vivo validation was limited to GRE‐based T_1_‐weighted contrasts due to SAR considerations. Given the sequence‐independent nature of the universal shim, subsequent research should evaluate its performance on cohorts with differing brain geometries and across a broader range of imaging contrasts relevant to neuroimaging research, including T_2_‐weighted, FLAIR, SWI, BOLD, and diffusion‐weighted imaging sequences.

## 5 Conclusion

This study presents the first successful implementation and in vivo validation of a universal B_1_^+^ shim solutions for human brain imaging at 10.5 T. Our findings, obtained with a limited yet representative participant cohort, demonstrate that the universal solution achieves comparable B_1_^+^ homogeneity and image quality to subject‐specific B_1_^+^ shimming for T_1_‐weighted imaging. Consequently, the universal B_1_^+^ shim solution offers a promising strategy to streamline UHF MRI workflows, significantly reducing examination time and enhancing imaging throughput without compromising image quality for fundamental neuroimaging applications.

## Funding

This work was supported by National Center for Research Resources (S10RR029672) and the National Institute of Biomedical Imaging and Bioengineering (P41EB027061, R01EB031787).

## Disclosure

This research was completed while Young Woo Park, Ph.D. was a staff researcher at the Center for Magnetic Resonance Research at the University of Minnesota. The content is solely the responsibility of the authors and does not necessarily represent the official views, opinions or policies of the National Institutes of Health, the Henry M. Jackson Foundation for the Advancement of Military Medicine Inc., the Uniformed Services University of the Health Sciences (USUHS), the Department of Defense (DoD), or the Departments of the Army, Navy, or Air Force. Mention of trade names, commercial products, or organizations does not imply endorsement by the U.S. Government.

## Supporting information


**Table S1:** Automated FreeSurfer cortical parcellation and subcortical segmentation results show significant (red, bold) differences around the peripheral regions of the brain such as temporal, occipital and parietal lobes as well as around the ventricles and the inferior regions of the brain such as basal ganglia and lateral ventricles. The P‐values were not adjusted for multiple comparisons. Abbreviations are as follows: WM—white matter; GM—gray matter.
**Table S2:**. Asymmetry Index of cortical and subcortical regions results show significant (red, bold) differences around peripheral and inferior regions of the brain such as temporal and occipital, lobes and cerebellar white matter. The P‐values were not adjusted for multiple comparisons.

## Data Availability

The data that support the findings of this study are available on request from the corresponding author. The data are not publicly available due to privacy or ethical restrictions.
