# Supplementary material for: 10.5 T In Vivo Head Imaging With Universal RF Shimming
Source: Magn Reson Med. 2026 Jan 19;95(5):2776–85. doi: 10.1002/mrm.70262 (PMC12962200; doi:10.1002/mrm.70262)
Supplement: Supplementary file 1 — Table S1: Automated FreeSurfer cortical parcellation and subcortical segmentation results show significant (red, bold) differences around the peripheral regions of the brain such as temporal, occipital and parietal lobes as well as around the ventricles and the inferior regions of the brain such as basal ganglia and lateral ventricles. The P‐values were not adjusted for multiple comparisons. Abbreviations are as follows: WM—white matter; GM—gray matter. Table S2:. Asymmetry Index of cortical and subcortical regions results show significant (red, bold) differences around peripheral and inferior regions of the brain such as temporal and occipital, lobes and cerebellar white matter. The P‐values were not adjusted for multiple comparisons. [file MRM-95-2776-s001.docx]

|  | **Volume (mm^3^)** | **Left Hemisphere** | | | **Right Hemisphere** | | |
| --- | --- | --- | --- | --- | --- | --- | --- |
|  |  | **Subj.** | **Univ.** | **P-value** | **Subj.** | **Univ.** | **P-value** |
| **Gray Matter** | **Frontal Lobe** | 65615 ± (10331) | 64710 ± (11374) | 0.457 | 65959 ± (12747) | 66067 ± (14464) | 0.940 |
|  | **Parietal Lobe** | 69265 ± (8183) | 65709 ± (10293) | **0.043** | 72308 ± (10509) | 68988 ± (11770) | **0.012** |
|  | **Temporal Lobe** | 44167 ± (6902) | 41101 ± (7112) | **0.013** | 42771 ± (5333) | 39624 ± (7014) | **0.012** |
|  | **Cingulate Lobe** | 11265 ± (1875) | 11128 ± (1848) | 0.725 | 9633 ± (1310) | 9406 ± (1665) | 0.299 |
|  | **Occipital Lobe** | 34876 ± (7169) | 33885 ± (7109) | 0.606 | 35168 ± (6640) | 32163 ± (7229) | **0.000** |
|  | **Insula Lobe** | 6310 ± (854) | 6604 ± (1005) | 0.283 | 6658 ± (382) | 6388 ± (541) | 0.323 |
| **White Matter** | **Frontal Lobe** | 63999 ± (8272) | 64445 ± (9580) | 0.501 | 62578 ± (8901) | 63016 ± (8914) | 0.428 |
|  | **Parietal Lobe** | 73698 ± (9922) | 73107 ± (10213) | 0.178 | 76111 ± (10558) | 75802 ± (11054) | 0.615 |
|  | **Temporal Lobe** | 31627 ± (3101) | 32992 ± (5335) | 0.350 | 30446 ± (3086) | 31625 ± (2497) | 0.198 |
|  | **Cingulate Lobe** | 15365 ± (1309) | 15308 ± (1454) | 0.730 | 13437 ± (1300) | 13292 ± (1061) | 0.242 |
|  | **Occipital Lobe** | 33702 ± (5799) | 36127 ± (7868) | 0.134 | 32153 ± (4488) | 30540 ± (4860) | **0.034** |
|  | **Insula Lobe** | 8680 ± (1071) | 8977 ± (1740) | 0.594 | 7780 ± (702) | 7378 ± (1236) | 0.310 |
| **Subcortical** | **Basal Ganglia** | 12348 ± (2036) | 12366 ± (1811) | 0.915 | 13968 ± (2485) | 13343 ± (1993) | **0.048** |
|  | **Cerebellar GM** | 57760 ± (8861) | 54159 ± (13809) | 0.219 | 56030 ± (8311) | 53922 ± (9124) | 0.260 |
|  | **Cerebellar WM** | 23001 ± (7838) | 21296 ± (6689) | 0.617 | 22749 ± (10027) | 27731 ± (4322) | 0.176 |
|  | **Thalamus** | 8523 ± (1307) | 8441 ± (1496) | 0.583 | 9911 ± (2048) | 8977 ± (1637) | 0.052 |
|  | **Hippocampus** | 4105 ± (411) | 4282 ± (352) | 0.384 | 4159 ± (338) | 3997 ± (504) | 0.436 |
|  | **Amygdala** | 1656 ± (156) | 1510 ± (282) | 0.368 | 1866 ± (341) | 2185 ± (262) | 0.078 |
|  | **Lateral Ventricles** | 18012 ± (3184) | 18056 ± (3199) | 0.826 | 15431 ± (2849) | 15816 ± (2819) | **0.029** |

**Supporting Table S1.** Automated FreeSurfer cortical parcellation and subcortical segmentation results show significant (**red, bold**) differences around the peripheral regions of the brain such as temporal, occipital and parietal lobes as well as around the ventricles and the inferior regions of the brain such as basal ganglia and lateral ventricles. The P-values were not adjusted for multiple comparisons. Abbreviations are as follows: WM – white matter; GM - gray matter

| **Volume Symmetry (L-R)/(L+R)** | | **Subj.** | **Univ.** | **P-value** |
| --- | --- | --- | --- | --- |
| **Gray Matter** | **Frontal Lobe** | 0.00 ± (0.02) | -0.01 ± (0.03) | 0.369 |
|  | **Parietal Lobe** | -0.02 ± (0.02) | -0.02 ± (0.01) | 0.489 |
|  | **Temporal Lobe** | 0.01 ± (0.03) | 0.02 ± (0.03) | 0.723 |
|  | **Cingulate Lobe** | 0.08 ± (0.06) | 0.08 ± (0.04) | 0.591 |
|  | **Occipital Lobe** | -0.01 ± (0.04) | 0.03 ± (0.02) | 0.204 |
|  | **Insula Lobe** | -0.03 ± (0.08) | 0.01 ± (0.06) | 0.249 |
| **White Matter** | **Frontal Lobe** | 0.01 ± (0.02) | 0.01 ± (0.02) | 0.883 |
|  | **Parietal Lobe** | -0.02 ± (0.01) | -0.02 ± (0.02) | 0.743 |
|  | **Temporal Lobe** | 0.02 ± (0.02) | 0.02 ± (0.04) | 0.917 |
|  | **Cingulate Lobe** | 0.07 ± (0.02) | 0.07 ± (0.01) | 0.763 |
|  | **Occipital Lobe** | 0.02 ± (0.04) | 0.08 ± (0.08) | **0.034** |
|  | **Insula Lobe** | 0.05 ± (0.05) | 0.10 ± (0.06) | 0.343 |
| **Subcortical** | **Basal Ganglia** | -0.06 ± (0.02) | -0.04 ± (0.02) | **0.002** |
|  | **Cerebellar GM** | 0.02 ± (0.06) | 0.00 ± (0.12) | 0.505 |
|  | **Cerebellar WM** | 0.02 ± (0.07) | -0.15 ± (0.15) | **0.044** |
|  | **Thalamus** | -0.07 ± (0.05) | -0.03 ± (0.07) | 0.157 |
|  | **Hippocampus** | -0.01 ± (0.03) | 0.04 ± (0.09) | 0.372 |
|  | **Amygdala** | -0.05 ± (0.10) | -0.18 ± (0.13) | 0.057 |
|  | **Lateral Ventricles** | 0.08 ± (0.08) | 0.07 ± (0.08) | 0.136 |

**Supporting Table S2**. Asymmetry Index of cortical and subcortical regions results show significant (**red, bold**) differences around peripheral and inferior regions of the brain such as temporal and occipital, lobes and cerebellar white matter. The P-values were not adjusted for multiple comparisons.
